# Electron Attachment and Electron Ionization of Formic Acid Clusters Embedded in Helium Nanodroplets

**DOI:** 10.1007/s13361-018-02124-z

**Published:** 2019-02-25

**Authors:** Masoomeh Mahmoodi-Darian, Linnea Lundberg, Samuel Zöttl, Paul Scheier, Olof Echt

**Affiliations:** 1 Department of Physics, Karaj Branch, Islamic Azad University, Karaj, Iran; 2 Institut für Ionenphysik und Angewandte Physik, Universität Innsbruck, Technikerstr. 25, A-6020 Innsbruck, Austria

**Keywords:** Formic acid, Cluster, Helium nanodroplets, Electron attachment, Resonance

## Abstract

We report the results of an experimental study of electron ionization of large helium nanodroplets doped with formic acid (FA). Several homologous series of cluster anions are observed, including [FA_n_-H]^−^, undissociated FA_n_^−^, and these ions complexed with one or more H_2_O. Some major features resemble those observed upon sputtering of frozen FA films but they differ significantly from results obtained by electron attachment to bare FA clusters in the gas phase. The FA_n_^−^ and (H_2_O)[FA_n_-H]^−^ series show abrupt onsets above *n* = 2 and 5, respectively. A prominent resonance in the anion yield occurs at 22.5 eV due to the formation of an intermediate He^−*^. Also observed are homologous series of [FA-H]^−^ or [FA_2_-H]^−^ complexed with helium. The cation chemistry is dominated by the production of protonated formic acid clusters, [FA_n_H]^+^, but various other homologous cluster ion series are observed as well.

**Electronic supplementary material:**

The online version of this article (10.1007/s13361-018-02124-z) contains supplementary material, which is available to authorized users.

**Figure:**
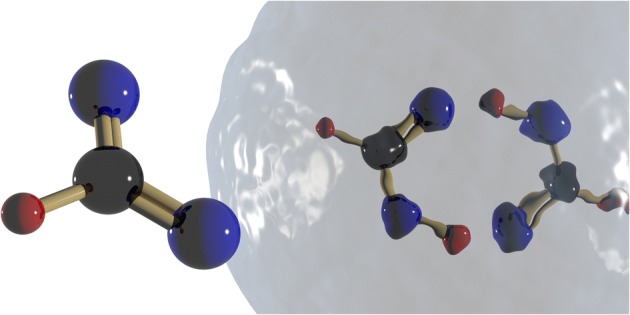
Graphical Abstract

## Introduction

Formic acid (HCOOH, abbreviated FA) is the simplest of the carboxylic acids of the form RCOOH in which R can be replaced by a number of substituents. Thus, FA serves as a model system for the properties of larger, more complex molecules. It plays a role in Earth’s atmospheric chemistry and has often been considered as a model for studying the biological systems exhibiting the organic acidic type of bonding [1]. It has been detected in the coma of comets and is probably present in interstellar nuclear ice [2]. Its abundance in interstellar ice exceeds that in interstellar gas by several orders of magnitude [3]; it may be key in the formation of amino acids such as glycine in the interstellar medium [4].

The FA dimer is a prototype for double bonded cyclic complexes; it has been studied extensively [5–7]. Infrared absorption spectra of FA clusters in noble gas matrices show that FA_3_ are chain-like [8], consistent with its polar character revealed in a molecular beam study utilizing an inhomogeneous electric field [9]. In the liquid bulk phase, however, X-ray and neutron diffraction indicate that FA molecules prefer to form short-branched hydrogen-bonded chains [10].

Charged clusters of FA have also received considerable attention. Mass spectra of ions desorbed from frozen films of FA bombarded by photons [3, 11], low-energy electrons [12, 13], energetic electrons and protons [14], and energetic heavy ions [4, 15, 16] have been recorded. Experimental studies of charged gas-phase clusters include photodissociation experiments [17, 18], UV-ionization [19] and electron attachment [20] to neutral clusters formed in a supersonic expansion, and mass spectrometry of ions formed in a liquid ionization [21] or variable pressure and temperature source [22].

Helium nanodroplets (HNDs) offer a unique environment to study chemical reactions at ultra-low temperatures [23, 24]. In a vacuum, HNDs cool by evaporation to 0.37 K; they are superfluid. Atoms and molecules that collide with a droplet are, for most systems, quickly incorporated into the droplet where they migrate freely. The excess energy released upon capture and aggregation of dopants is quickly removed by evaporation of weakly bound He atoms (binding energy 0.62 meV in bulk helium). Electron bombardment of doped droplets leads to various intermediates, including He^+^, electronically excited He^*^ and He^*−^, and electron bubbles. These species may migrate (or resonantly hop) to the dopant and transfer their energy and/or charge, resulting in negative or positive dopant ions [25–28].

The goal of the present work is to study the interaction of electrons with formic acid clusters embedded in HNDs, and to compare the composition and yield of anions and cations thus produced with those obtained by the interaction of electrons or photons with bare gas-phase FA clusters [19, 20], of energetic heavy ions with frozen FA films [15, 16], and ions extracted from high-pressure sources [22].

## Experimental

HNDs were produced by expanding helium (Linde, purity 99.9999%) at a stagnation pressure of about 20 bar through a 5-μm nozzle, cooled by a closed-cycle cryostat (Sumitomo Heavy Industries LTD, model RDK-415D) to about 9.5 K for positive and 9.4 K for negative ions into a vacuum. Droplets formed at these conditions contain an average of about 5 × 10^5^ atoms [29]. The resulting supersonic beam was skimmed by a 0.8-mm conical skimmer, located 8-mm downstream from the nozzle and passed through a 20-cm-long pick-up region into which FA (Sigma-Aldrich, 98–100%) was introduced. Partial FA pressures were 2.4 × 10^−6^ and 1.6 × 10^−6^ mbar for positive and negative ion experiments, respectively.

The doped helium droplets passed through a differentially pumped vacuum chamber where they were crossed with an electron beam of variable energy. Anions were formed at 22.5 eV whereas cations were formed at 99 eV. The electron energy scale was calibrated via the 22 eV resonance leading to the formation of He*^−^ [26, 30]. Ions were accelerated into the extraction region of a commercial orthogonal time-of-flight mass spectrometer equipped with a reflectron (Tofwerk AG, model HTOF). For both anions and cations, the effective mass resolution was *m*/Δ*m* = 3000 (Δ*m* = full-width-at-half-maximum). The base pressure in the mass spectrometer was 2.6 × 10^−7^ and 2.9 × 10^−7^ mbar for negative and positive ions, respectively. The ions were detected by a micro-channel plate operated in single ion counting mode and recorded via a time to digital converter. Additional experimental details have been described elsewhere [31].

Mass spectra were evaluated by means of a custom-designed software [32]. The abundance of ions is derived from the mass spectra by a matrix method. The routine corrects for experimental artifacts such as background signal levels, imperfect peak shapes, and mass drift over time, and it takes into account the isotope pattern of all relevant elements. Hydrogen, helium, and oxygen are very nearly monoisotopic but contributions from ions containing one or more ^13^C (natural abundance 1.07%) can be significant.

## Results and Discussion

### Negative Ion Mass Spectra

**Figure 1:**
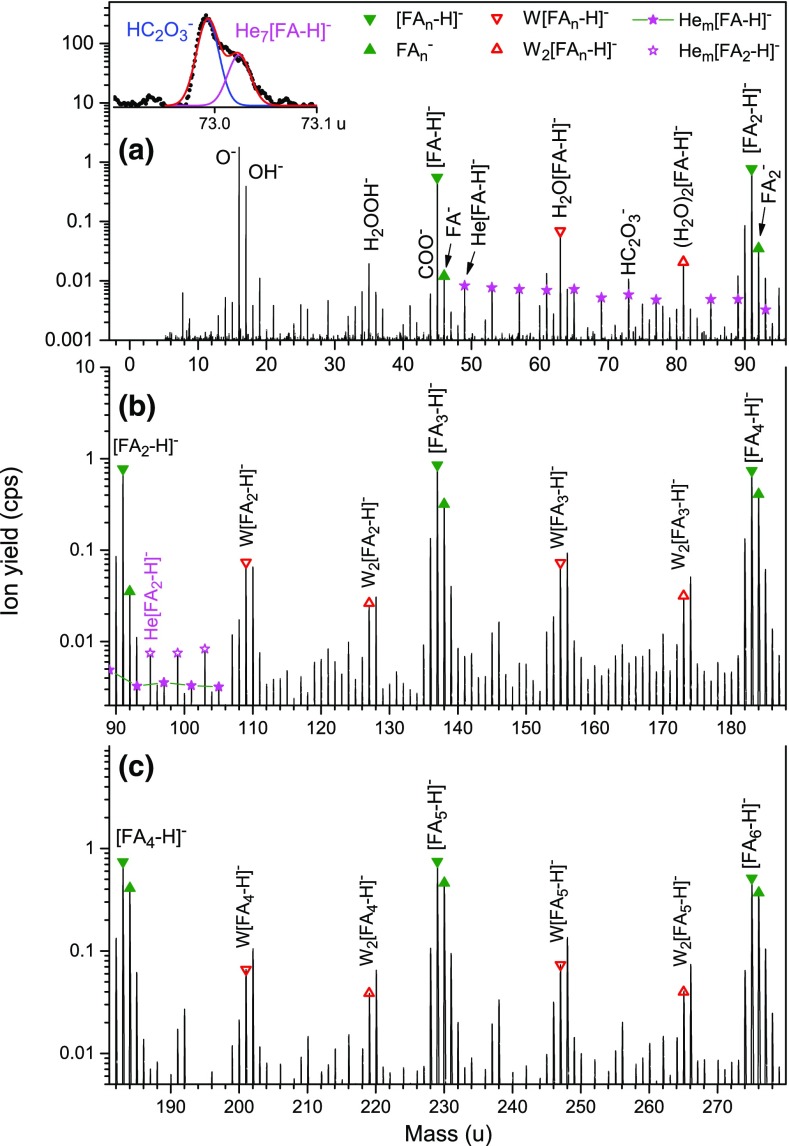
Negative ion mass spectrum of helium nanodroplets (HNDs) doped with formic acid (FA, HCOOH) obtained by electron attachment at 22.5 eV. Panel (**a**) covers the mass region up to the FA dimer, panel (**b**) covers the dimer to tetramer, and panel (**c**) the tetramer to hexamer. The most abundant anions are due to dehydrogenated or undissociated FA clusters (closed triangles). Other prominent ion series are due to [FA_n_-H]^−^ or FA_n_^−^ with one or two water (W) attached (open triangles). Also observed are [FA-H]^−^ and [FA_2_-H]^−^ complexed with helium. The inset in panel a demonstrates that He_7_[FA-H]^−^ ions (and most other He_m_[FA-H]^−^ ions) can be resolved from other anions

Figure 1 shows sections of a negative ion mass spectrum obtained by attaching electrons at 22.5 eV to helium nanodroplets (HNDs) doped with formic acid (FA). The panels cover different but overlapping mass regions: up to the FA dimer, from the dimer to the tetramer, and from the tetramer to the hexamer (panels a, b, and c, respectively).

The most abundant anions appearing at and below 46 u, the mass of FA, are O^−^, OH^−^, and HCOO^−^ (labeled [FA-H]^−^). Also observed are H_2_OOH^−^, COO^−^, and HCOOH^−^. Note, though, that the mass peak at 46 u (labeled FA^−^) has a significant (≈ 50%) contribution from H^13^COO^−^. O^−^, OH^−^, and HCOO^−^ have been detected upon electron attachment to gas-phase FA monomers but their abundance depends greatly on the electron energy [20, 33, 34]. HCOO^−^ is the most abundant; it is formed via a shape resonance around 1.25 eV. Using isotopically labeled FA (DCOOH and HCOOD), Martin et al. [20] could show that hydrogen abstraction from gas-phase FA almost exclusively operates from the OH site, producing a negatively charged hydrocarboxyl radical HCOO which has a large (3.50 eV [35]) electron affinity. Less abundant anions observed in previous gas-phase studies are OH^−^ formed around 7.5 eV via a core-excited shape resonance, and O^−^ formed above 8 eV [33]. The thermodynamic thresholds for the formation of these ions from FA + e^−^ are 3.48 and 3.84 eV, respectively [33].

H^−^ has been observed around 7.5 eV by Prabhudesai et al. [34]; presumably, it escaped detection in other reports because it is emitted with high kinetic energy.

The weaker peaks in Figure 1a, namely H_2_OOH^−^, COO^−^, and HCOOH^−^, were not detected upon electron attachment to gas-phase FA but they have been identified upon bombarding frozen films of FA with energetic (65 MeV) fission fragments emitted from radioactive ^252^Cf [16]. Long-lived FA^−^ cannot be formed by attachment to gas-phase FA monomers without collisional stabilization. Furthermore, the adiabatic electron affinity of FA is negative, i.e., the extra electron would be unbound [33]. An ab initio calculation with the 6-311 ++G** basis set by Ziemczonek and Wroblewski yielded an adiabatic electron affinity (AEA) of − 1.27 eV for FA^−^ [36]. Valadbeigi and Farrokhpour obtained similar values (within ± 7%) using CBS-Q, G4MP2, W1BD, and G2MP2 methods [37].

Low-energy electron-induced desorption from thin films of FA provides another venue to form anions. In those studies, the most intense anion is H^−^ with a resonance at 9.5 eV; O^−^, OH^−^, and HCOO^−^ have successively weaker yields with onsets around 8 eV [12, 13]. H^−^ is the favored desorption product because it receives the major share of the kinetic energy release thanks to its small mass.

We now turn to cluster anions. The most abundant series of mass peaks is due to [FA_n_-H]^−^, marked in Figure 1 by full triangles pointing down. Mass ranges in panels a, b, and c have been chosen such that homologous ion series line up. Even-numbered members of [FA_n_-H]^−^ appear to the far left and far right in each panel; odd-numbered members appear near the center.

Another ion series is labeled FA_n_^−^ (full triangles pointing up). The mass peak labeled FA_2_^−^ has a significant (≈ 50%) contribution from [FA_2_-H]^−^ ions that contain one ^13^C but for higher members of the FA_n_^−^ series the contribution from ions containing ^13^C is below 10%.

Other prominent mass peaks in Figure 1b and c, marked by open triangles, are due to [FA_n_-H]^−^ with one or two H_2_O (abbreviated W) attached. Mass peaks due to W[FA_n_-H]^−^ are followed by peaks due to W[FA_n_]^−^. Contributions to these peaks from dehydrogenated ions containing ^13^C are negligible; for *n* ≥ 3, the abundance of W[FA_n_]^−^ even exceeds that of W[FA_n_-H]^−^. Similarly, mass peaks due to W_2_[FA_n_-H]^−^ are followed by more intense mass peaks due to W_2_[FA_n_]^−^.

A weaker series of mass peaks is due to He_m_[FA-H]^−^ and He_m_[FA_2_-H]^−^; they are flagged in panels a and b by asterisks. Some members of the He_m_[FA-H]^−^ series are only partly resolved as illustrated in the inset in panel a. The peak due to He_7_[FA-H]^−^ appears at 73.01585 u, 0.023 u above another mass peak that we assign to an ion with the stoichiometry HC_2_O_3_^−^ (no other reasonable combination of H, C, O will produce an ion at a nominal mass of 73 u). Other members of the He_m_[FA-H]^−^ series can be identified similarly up to *m* = 15, with the exception of *m* = 9 which coincides with W_2_[FA-H]^−^.

**Figure 2:**
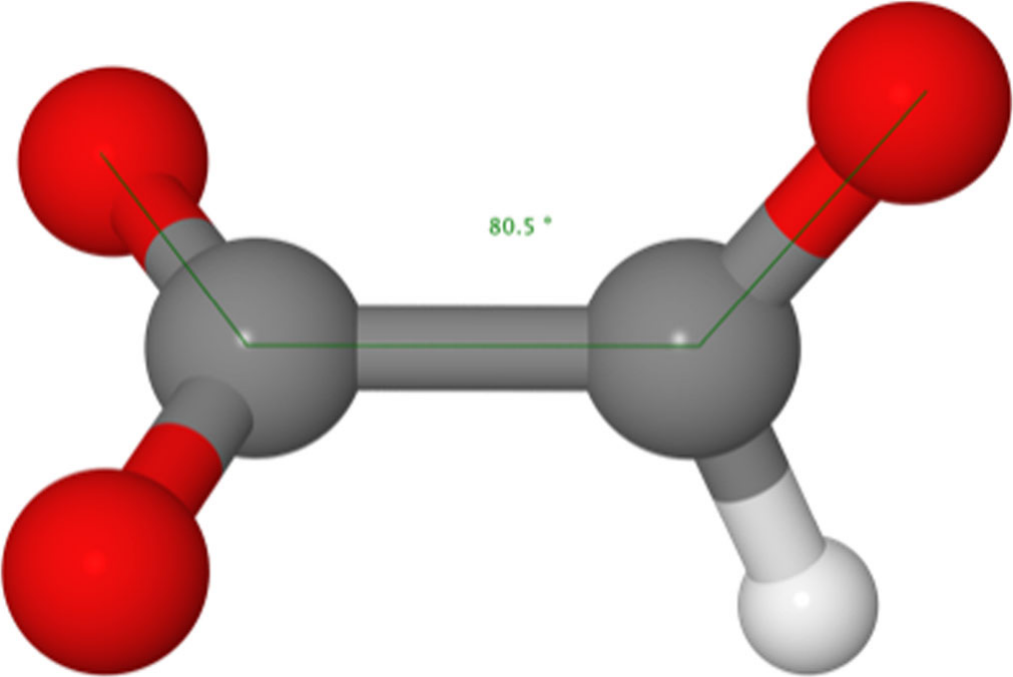
The putative ground state structure of the HC_2_O_3_^−^ anion

The ions mentioned above form homologous series that are identified for 1 ≤ *n* ≤ 15 and beyond. There is only one ion of significant abundance in the region between the FA monomer and dimer that does not give rise to a homologous series, namely HC_2_O_3_^−^ at 73 u. We could not find any information about this anion in the literature. We have computed its structure with the density functional B3LYP with basis set aug-cc-pvtz. The putative ground state structure is displayed in Figure 2; it is nearly 2 eV lower in energy than any of the other isomers that were tested. The bond between the two carbons is covalent and the bond length is about 1.53 Å. The coordinates of all atoms are provided as Electronic Supplementary Material.

**Figure 3:**
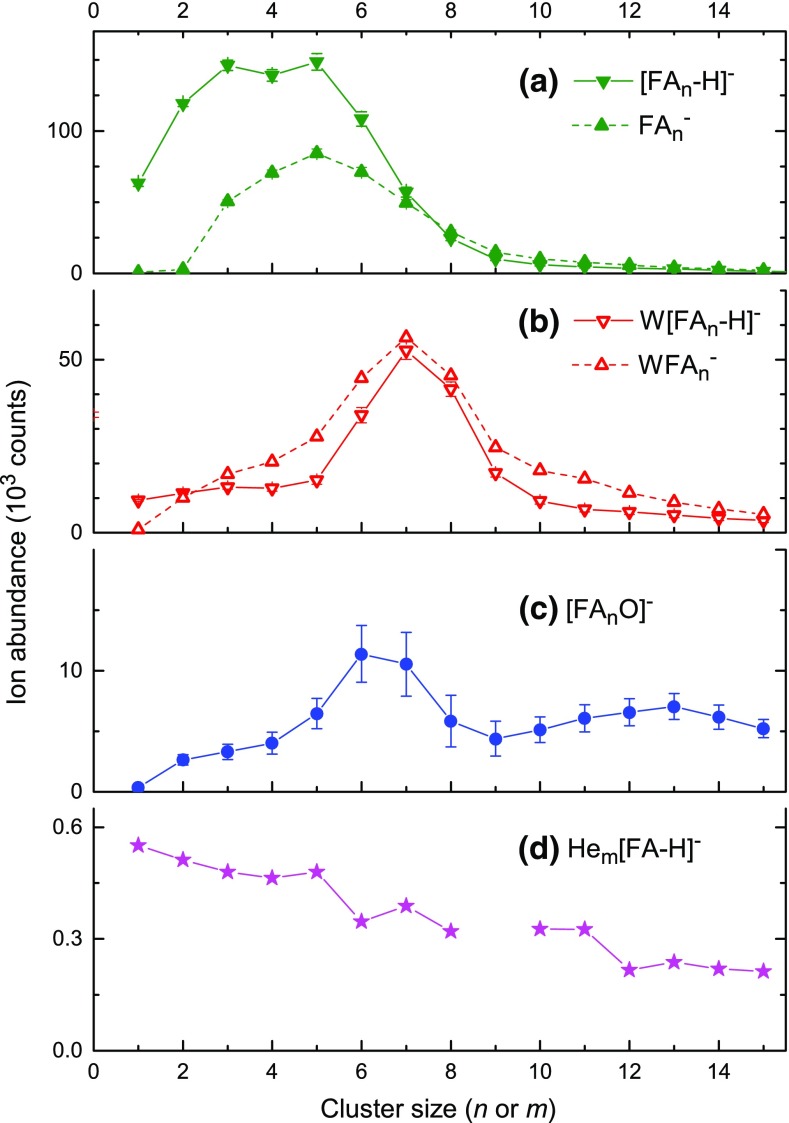
Abundance distributions of cluster anion series extracted from the spectrum in Figure 1 taking into account isotopologues and impurities. FA denotes formic acid; W denotes water

The abundance distributions of select ion series are derived from the mass spectrum in Figure 1 by a custom-designed software that takes into account all possible isotopologues, and contributions from impurities, background, and isotopologues of other ions [32]. Results are compiled in Figure 3. Error bars are displayed in panels a through c; most of them are smaller than the symbol size. Note the linear ordinate as opposed to the logarithmic ordinate in Figure 1.

The [FA_n_-H]^−^ series shown in Figure 3a rises slightly with increasing size *n*, reaches a plateau from *n* = 3 to 5, and then drops off strongly. The FA_n_^−^ series rises abruptly above *n* = 2 and peaks around *n* ≈ 5. The abrupt onset in the ion abundance of FA_n_^−^ beyond *n* = 2 is consistent with the above-mentioned work by Ziemczonek and Wroblewski [36]. The authors obtained − 1.16 eV and − 0.09 eV for the AEAs of the dimer and trimer in their putative ground state structures. Although the vertical detachment energies of the corresponding (structurally relaxed) anions were not computed, the near-zero AEA of FA_3_ combined with a detachment barrier may result in a substantial positive vertical detachment energy of FA_3_^−^, i.e., a long-lived anion. On the other hand, the large negative AEA of FA_2_ probably renders the FA_2_^−^ anion short-lived.

In Figure 3b, the W[FA_n_-H]^−^ series rises abruptly above *n* = 5 and peaks around *n* ≈ 7. The yield of the WFA_n_^−^ series exceeds that of the dehydrogenated series above *n* = 2. The four ion series displayed in Figure 3a and b reach the same abundance at *n* = 7. The [FA_n_O]^−^ series (Figure 3c) is much weaker.

Anions of FA clusters have been formed previously by two different approaches. Andrade et al. identified two anion series, namely [FA_n_-H]^−^ and W[FA_n_-H]^−^ for 1 ≤ *n* ≤ 10 upon bombardment of FA cryofilms with fission fragments [15, 16]. The size distribution of W[FA_n_-H]^−^ showed an abrupt onset above *n* ≈ 5 similar to the one seen in Figure 3b. In their work, [FA_n_-H]^−^ and W[FA_n_-H]^−^ become equally abundant around *n* = 9. Visual inspection of their mass spectrum (Figure 4 in [16]) indicates the presence of W_2_[FA_n_-H]^−^ starting at *n* = 7. FA_n_^−^ may have been formed as well but would have escaped detection because of low mass resolution.

**Figure 4:**
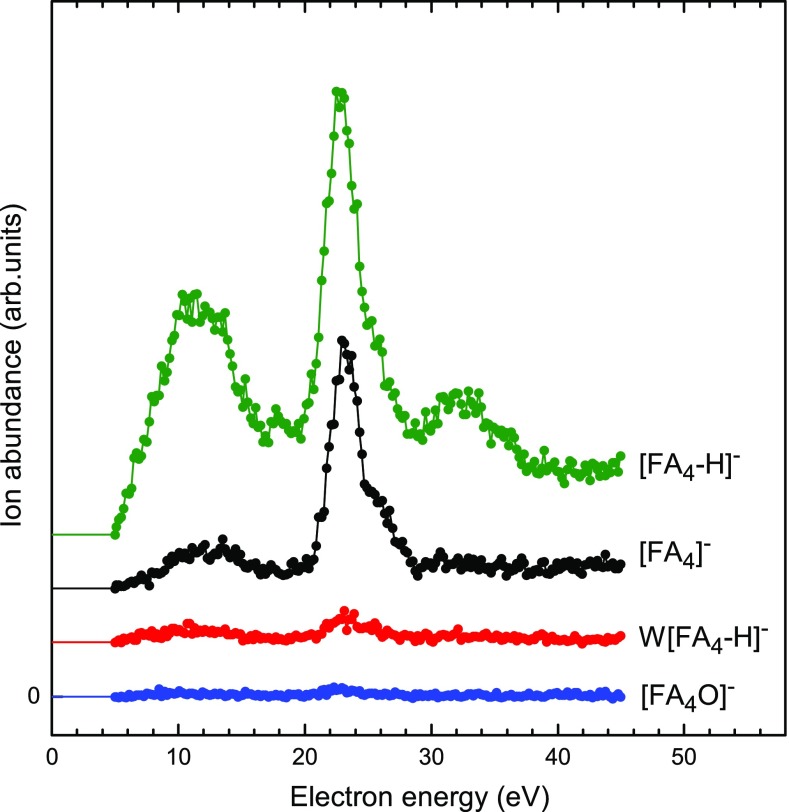
Dependence of the anion abundance on the electron energy for anions containing the FA tetramer. Data are stacked; the trace below 5 eV indicates the baseline

Martin et al. have attached electrons to bare FA clusters formed by adiabatic expansion of FA seeded in helium [20]. Their published mass spectrum, acquired at an electron energy of 1 eV (just below the [FA-H]^−^ resonance), extends to 200 u. Similar to our data, they observe predominantly [FA_n_-H]^−^ and FA_n_^−^. FA^−^ and FA_2_^−^ are very weak but the two series reach nearly the same abundance at *n* = 4. The fact that, in our data, the two series reach about the same abundance at *n* = 7, and even later in the ion desorption experiments by Andrade et al. [15, 16] suggests that the two series have different neutral precursors, i.e., that the average number of FA monomers lost upon anion formation is different. However, the different methods in which the charged clusters are formed may play a role as well.

Other features in the mass spectrum reported by Martin et al. [20] differ markedly from our data. Between the monomer and dehydrogenated dimer ion (i.e., between 46 and 91 u), they observe no ions other than W[FA-H]^−^ even though the signal-to-noise ratio in this region is 0.1% relative to the [FA_2_-H]^−^ peak. In the region above the FA dimer anion, W[FA_2_-H]^−^ and W[FA_3_-H]^−^ are absent; their yield must be less than 0.2% of the corresponding water-free ions. Instead, a pair of intense ions appear at 105 and 113 u, i.e., 13 u and 21 u above FA_2_^−^, and another pair at 151 and 159 u, i.e., 13 and 21 u above FA_3_^−^ [38]. The mass peaks at 151 and 159 u in Martin’s spectrum are followed by nearly equally intense satellites at 152 and 160 u. The stoichiometry of these six anions above FA_2_^−^ (i.e., at mass 105, 113, 151, 152, 159, and 160 u) remains unclear but it is worth mentioning that the discussion in [20] is affected by an invalid mass assignment [38]. There is no evidence for any of these ions in our mass spectrum; their yield would be well below 1% relative to that of the main peaks, [FA_n_-H]^−^.

It is difficult to assess the presence and origin of potential H_2_O impurity in the doped HNDs. Large HNDs tend to pick up residual gas, including water vapor, on their path from droplet formation to the ionizer. Water might also be present in the sample that is introduced into the pick-up cell. Water and FA have nearly the same vapor pressure (their boiling points differ by only 0.8 °C); hence, the standard procedure of freezing out water vapor in the sample inlet cannot be used. We observe an even larger propensity for mixed FA-water clusters in positive ion mass spectra, see the “Positive Ion Mass Spectra” section.

However, the detection of W[FA_n_-H]^−^ does not necessarily imply the presence of H_2_O in the neutral HND. Baptista et al. have studied the structure, energetics, and temporal behavior of W[FA_n_-H]^−^, *n* ≤ 4, by Born-Oppenheimer molecular dynamics [39]. They suggest that these anions arise upon sputtering of frozen FA films from the reaction.

1 $$ {\left(\mathrm{HCOOH}\right)}_{\mathrm{n}}\to {\left(\mathrm{HCOOH}\right)}_{\mathrm{n}-1}{\mathrm{OH}}^{-}+{\mathrm{HCO}}^{+} $$

and that hydrogen abstraction from FA by the hydroxyl anion leads to a distinct water moiety, i.e., to anions of the form (HCOOH)_n-2_(H_2_O)HCOO^−^.

What else, other than a possible water contamination, may cause the stark difference between our spectrum and the one reported by Martin et al. [20]? Martin et al. recorded their negative ion mass spectrum at 1 eV while ours was recorded at 22.5 eV. Another factor could be the different cluster environment (bare clusters versus clusters embedded in a HND). The helium environment has several effects [24], including a change in the electron energy, different vibrational temperatures (roughly 200 K for bare FA clusters [40] versus 0.37 K for HNDs [23]), and a possible suppression of intra- or intermolecular dissociation channels [41].

In order to shed light on the effect of electron energy, we have recorded mass spectra between 5 and 45 eV. Figure 4 displays the abundance of [FA_4_-H]^−^, FA_4_^−^, W[FA_4_-H]^−^, and [FA_4_O]^−^ versus electron energy. All ions show a main resonance around 22.5 eV. [FA_4_-H]^−^ displays additional maxima near 12 eV and 32 eV; these features are less prominent for the other ions. The resonance at 22.5 eV is a characteristic feature of doped HNDs, due to resonant formation of electronically excited metastable He*^−^ anions. These ions are highly mobile in helium; they will be attracted towards the dopant by ion-induced dipole interaction followed by electron transfer to the dopant [26, 27]. Therefore, it is not surprising that all anions shown in Figure 4 exhibit this resonance. Furthermore, large HNDs suppress anion formation at low electron energies because of the formation of electron bubbles and the reduced ejection probability of dopant anions [28].

The broad maximum around 12 eV is probably characteristic of FA. It has been shown that intramolecular dissociation upon electron attachment can be strongly suppressed when the molecules are embedded in HNDs [41]. It is thus conceivable that the products of electron attachment to FA films (namely H^−^, O^−^, OH^−^, and HCOO^−^ with resonances at 9.5 eV, 10 eV and higher, 11.3 eV and higher, and 12.7 eV and higher [13]) contribute to the broad resonance at 12 eV observed here for [FA-H]^−^. Moreover, the HND shifts the energy scale by some 1.5 eV to lower values because the bottom of the conduction band in condensed helium lies above the vacuum level [24]. That said, we note that the yield of OH^−^ in Figure 1a is no less than that of [FA-H]^−^, i.e., there is no evidence for suppression of electron-induced dissociation in the present situation. Unfortunately, we were not able to acquire data below 5 eV, in the region of the [FA-H]^−^ resonance in gas-phase experiments.

We conclude with a discussion of anions complexed with helium. The anion mass spectrum (Figure 1) reveals the presence of He_m_[FA-H]^−^ and He_m_[FA_2_-H]^−^. The abundance of He_m_[FA-H]^−^ versus size *m* is displayed in Figure 3d [42]. There seem to be distinct anomalies (abrupt drops) at *m* = 5 and 11 (and perhaps also at *m* = 7) which may indicate local anomalies (magic numbers) in the dissociation (evaporation) energy. Anomalies in the size distribution (and stability) of He_m_M^±^ ions, where M is some atomic or molecular ion, have been observed for many systems (for a recent compilation see Table 4 in [24]). They often correlate with closure of solvation shells, subshells, or second or even third solvation shells [43–45]. An interesting situation arises if M^±^ is a planar ion, e.g., coronene; the approximate structure of coronene cluster ions has been inferred from magic numbers in the abundance distributions of He_m_M_2_^+^, He_m_M_3_^+^, and He_m_M_4_^+^ [46].

The statistical significance of anomalies in the distribution of He_m_[FA-H]^−^ requires further experimental confirmation but it is interesting to compare the present results with a study of electron attachment to HNDs doped with acetic acid (CH_3_COOH, abbreviated AA) by da Silva et al. [47]. The authors detected complexes of dimer anions and larger clusters (mostly AA_n_^−^ and some [AA_n_-H]^−^) with helium attached, but no helium was found to be attached to the dehydrogenated monomer anion [AA-H]^−^. The authors conjectured that the negative excess charge in [AA-H]^−^ is unfavorable for attachment of helium while the *n*-1 neutral acetic acid moieties in cluster ions bind helium more strongly [47]. No related effect is observed in the present study; the yield of He_m_[FA-H]^−^ even exceeds that of He_m_[FA_2_-H]^−^. One factor that may contribute to these different observations may be the size of the neutral HNDs which averaged 10^4^ atoms in the study of acetic acid as opposed to about 5 × 10^5^ in the present study.

### Positive Ion Mass Spectra

**Figure 5:**
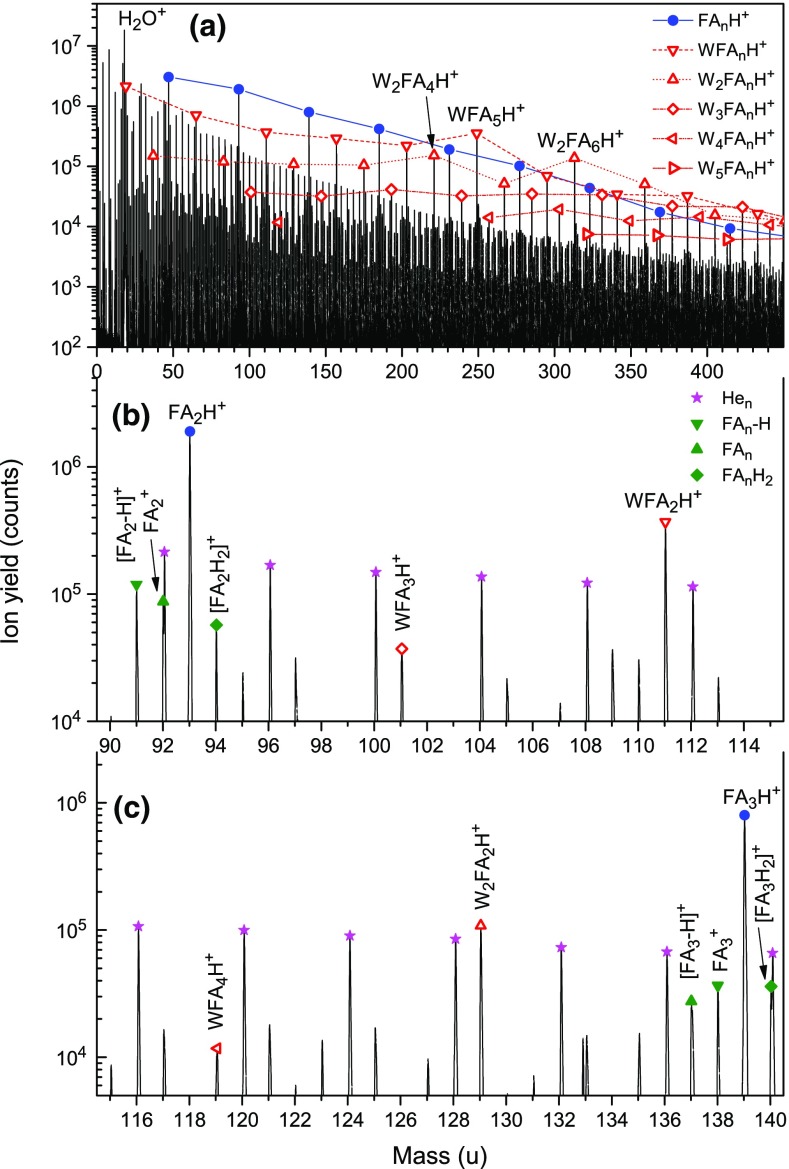
Positive ion mass spectrum of HNDs doped with formic acid recorded at an electron energy of 99 eV. The most abundant ions are flagged in panel (**a**); they are due to protonated FA_n_ and protonated FA_n_ with one or more water (W) attached. Additional, weaker cluster ion series are identified in panels (**b**) and (**c**) which cover the mass region between the FA dimer and trimer

Figure 5a presents a mass spectrum of positive ions formed by electron ionization of FA clusters embedded in HNDs recorded at electron energy of 99 eV. The most prominent mass peaks are marked; they are due to protonated FA cluster ions (full dots, labeled FA_n_H^+^), and protonated FA cluster ions containing water molecules (open symbols, labeled W_m_FA_n_H^+^ where *m* ≤ 5). The series of closely spaced mass peaks is due to He_n_^+^.

Figure 5b and c zoom into the region between the FA dimer and trimer. All mass peaks at and above the 1% level of the main FA_n_H^+^ mass peaks are identified. In addition to the ions mentioned above, these peaks arise from [FA_n_-H]^+^, FA_n_^+^, and [FA_n_H_2_]^+^. Although some of these ions have the same nominal mass as He_n_^+^ (marked by asterisks), they are resolved in the mass range shown. For example, the mass difference between He_23_^+^ and FA_2_^+^, both at a nominal mass of 92 u, is 0.049, well within the resolving power of the instrument.

There are noticeable differences, but also some agreements, between our positive ion mass spectra and those reported previously. Feng and Lifshitz formed FA cluster ions by electron impact in a temperature and pressure-variable source [22]. Apart from protonated [FA_n_H]^+^ and W_m_[FA_n_H]^+^ (*m* ≤ 2), they observed weak signals due to unprotonated cluster ions [FA_n_]^+^ and dehydrogenated cluster ions [FA_n_-H]^+^, in agreement with our data.

Bernstein and coworkers formed neutral FA clusters in a supersonic expansion of FA seeded in helium; clusters were ionized by photons at 26.5 eV [19]. Spectra were also measured for mixtures of FA and water. In addition to prominent [FA_n_H]^+^ and W_m_[FA_n_H]^+^ (*m* ≤ 4), they observed a cluster ion series at 14 u above the mass of [FA_n_H]^+^ with a yield of about 10% of the [FA_n_H]^+^ series. The ions were attributed to [FA_n_CH_2_H]^+^, thought to result from loss of O_2_ from [FA_n_H]^+^. There is no evidence for these ions in our data (see Figure 5b; [FA_2_CH_2_H]^+^ would appear at 107 u). Bernstein and coworkers also observed FA_2_^+^ but no larger unprotonated FA cluster ions.

Andrade et al. reported positive and negative ion mass spectra of FA cluster ions formed by bombarding frozen films of FA with fission fragments emitted from radioactive ^252^Cf [15, 16]. The main cluster ion series were due to [FA_n_H]^+^ and W_m_[FA_n_H]^+^ (*m* ≤ 3). Another ion series, 14 u above the mass of [FA_n_H]^+^, was assigned to [FA_n-1_CO_3_ H]^+^ (rather than [FA_n_CH_2_ H]^+^ as in the work by Bernstein and coworkers [19]). A second minor ion series, at 32 u above the mass of [FA_n_H]^+^, was attributed to [FA_n_O_2_ H]^+^. Our data show no evidence for these ions at the 1% level.

**Figure 6:**
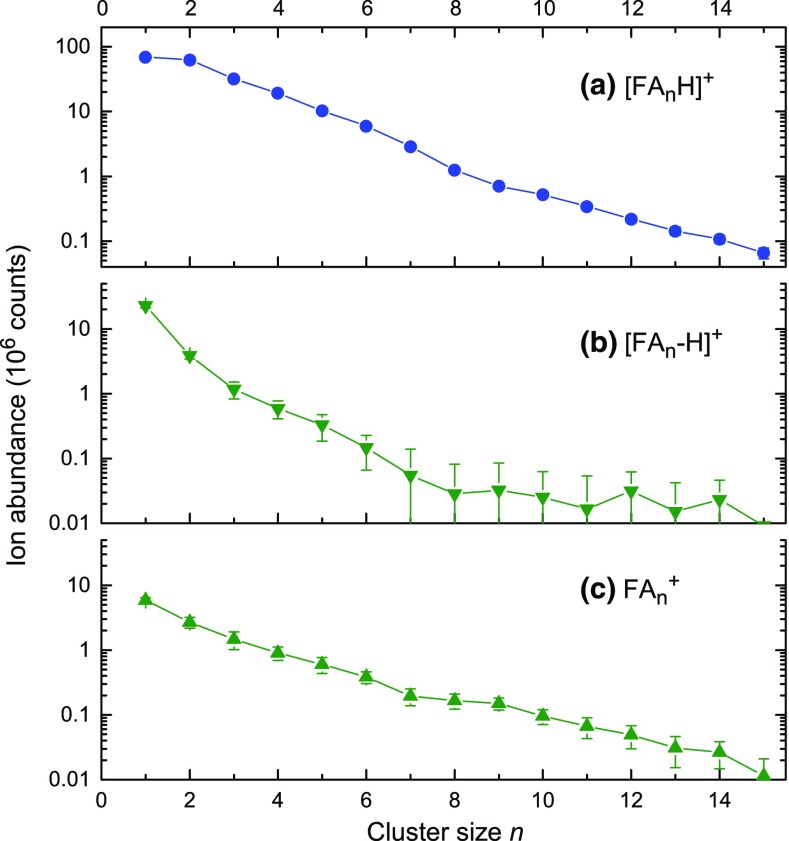
Abundance distributions of cluster cation series extracted from the spectrum in Figure 5

Figure 6 compiles the abundance distributions of [FA_n_H]^+^, [FA_n_-H]^+^, and [FA_n_]^+^. The distributions of [FA_n_H]^+^ and [FA_n_-H]^+^ reveal no anomalies within the experimental uncertainties (error bars in Figure 6 are smaller than the symbol sizes). The [FA_n_]^+^ distribution displays very small stepwise decreases at *n* = 6 and 9 that may be statistically significant.

Anomalies previously reported for the distributions of protonated FA cluster ions are surprisingly inconsistent. Andrade et al. report a smooth, exponentially decreasing distribution for *n* ≤ 7 upon sputtering of frozen FA films [16], in agreement with our results. Feng and Lifshitz mention a pronounced maximum at [FA_6_H]^+^ formed in a temperature and pressure-variable ion source [22]. Bernstein and coworkers, employing single-photon ionization of FA clusters formed in a supersonic beam, observe a magic number at [FA_5_H]^+^, although only under certain experimental conditions [19].

The differences might be due to a combination of factors, including poor signal-to-noise ratios, and differences in the ion formation process (another possible factor, namely size-dependent detection and ionization efficiencies, is less likely to cause abrupt changes in the ion abundance). Inspection of mass spectra reported by Feng and Lifshitz reveals a “magic character” not only for [FA_6_H]^+^ but also for [FA_5_H]^+^ or [FA_4_H]^+^, depending on the temperature and pressure of the thermal cluster ion source [22]. Bernstein and coworkers detect the emergence of magic [FA_5_H]^+^ if the extraction of ions from the ion source is delayed by some 220 us, thus allowing for slow unimolecular dissociation of [FA_n_H]^+^ into [FA_n-1_H]^+^ or even [FA_n-2_H]^+^ [19]. The authors assume that single-photon ionization of FA clusters does not provide sufficient excess energy for prompt intermolecular fragmentation although the difference between vertical and adiabatic ionization energies would suffice to initiate proton transfer and subsequent loss of COOH. For short time delays between ion formation and extraction, the distribution of [FA_n_H]^+^ will therefore be smooth, reflecting the smooth distribution of neutral FA_n_; for large delays, a magic number emerges because this ion, presumably, features enhanced stability.

The fact that magic numbers in mass spectra may become more pronounced with increasing time after ionization is well established [48, 49]. Conceivably, electron ionization of clusters embedded in HNDs might suppress fragmentation and the concomitant evolution of magic numbers that relate to the stability of ions. Past experiments, however, indicate that mass spectra of van der Waals clusters embedded in HNDs are very similar to those obtained from bare clusters [50]. The seemingly different results reported for [FA_n_H]^+^ (no magic number in our spectra and those of Andrade et al. [16], a magic number in Bernstein’s spectra [19], changing magic numbers in Lifshitz’s spectra) are probably caused by the *weakness* of the magic number character of [FA_5_H]^+^. In Figure 3 of ref. [19], its ion yield is only 20% larger than that of the average yield of [FA_4_H]^+^ and [FA_6_H]^+^. Evaporation energies of [FA_n_H]^+^, calculated for *n* ≤ 7 with the Gaussian 92 package at various levels of theory do, indeed, show no clear evidence for enhanced stability of [FA_5_H]^+^ [51, 52, 53–56].

An interesting computational result, though, possibly related to the reported anomaly at [FA_5_H]^+^, is a structural change from open-ended chain structures for *n* ≤ 5 to chain structures terminated by cyclic FA dimers at both ends for *n* = 6, 7 [18, 51, 54, 57, 58]. These results are supported by infrared photodissociation spectroscopy which reveal the presence of free OH groups at both ends of the chain for *n* ≤ 5, but not for *n* = 7 [18]. The structural change correlates with a change in the unimolecular dissociation pattern, namely from preferential evaporation of FA monomers for *n* ≤ 5 to FA dimers for *n* ≥ 6 [19, 22].

We are not aware of any experimental work related to the stability of [FA_n_-H]^+^ or [FA_n_]^+^; these ions were either not observed beyond *n* = 2 [16, 19], or not evaluated [22].

Finally, we address the appearance of mixed, protonated FA-water cluster ions, W_m_[FA_n_H]^+^. Their yield is large as seen in Figure 5a where they are marked for *m* = 1, 2, 3, 4, 5. Bernstein and coworkers observed that the yield of W[FA_n_H]^+^ does not change upon addition of small amounts of water to the FA vapor in the supersonic expansion source; they concluded that W[FA_n_H]^+^ arises from [FA_n + 1_H]^+^ via loss of a CO molecule [19]. Water is a notorious contaminant in mass spectra of doped helium nanodroplets, partly because large droplets have a large cross-section for picking up water molecules on their path from the supersonic source to the ionizer. However, the amount of water in the present spectra is unprecedented. Past experiments involving HNDs doped with clusters of C_60_, hydrocarbons, or metal atoms rarely show the presence of more than one or two water molecules [24]. Furthermore, the abundance of FA cluster cations containing multiple water molecules relative to water-free cluster cations is much larger than for anions. We do not see any reason why a possible water contamination in the background gas, the helium droplets, or the formic acid sample would have been different during these measurements. Rather, the difference appears to be due to different ionization efficiencies or intracluster ion-molecule reactions. More work is needed to pin down the exact mechanism.

The W[FA_n_H]^+^ series exhibits a pronounced local maximum at *n* = 5 in agreement with previously reported mass spectra obtained from a thermal ion source [22], photoionization [19], or electron ionization [17] of a supersonic beam, sputtering by fission fragments [15], and liquid ionization mass spectrometry [21]. The consistent observation of a magic number in the W[FA_n_H]^+^ ion series at *n* = 5 (as opposed to the lack of consistency for the allegedly magic [FA_5_H]^+^) reflects the strength of its magic number character: in our data, the abundance of W[FA_5_H]^+^ is 2.5 times the average yield of the adjacent peaks in the homologous series, while the yield of [FA_5_H]^+^ (in the spectrum reported by Bernstein and coworkers [19]) is merely 1.2 times the average of the adjacent homologous ions.

The structure of W[FA_5_H]^+^ has been the subject of several theoretical investigations; they suggest that the ion has a ring structure composed of five FA molecules with one water molecule located inside the ring [17, 21, 54]. The conclusion has been confirmed by infrared spectroscopy [17]. Baptista et al. have investigated [FA_n_H]^+^ and W[FA_n_H]^+^ by Born-Oppenheimer molecular dynamics, though only for *n* ≤ 4 [58]. An interesting result is the importance of H migration between the FA moieties for the cluster stability. Furthermore, cluster growth does not present a regular pattern of nucleation; there is no tendency to form a solvation shell around a distinct ion.

The W_2_[FA_n_H]^+^ ion series exhibits local maxima at *n* = 4 and 6 (Figure 5a) in agreement with data reported by Feng and Lifshitz [22]; to the best of our knowledge, no computational work has been reported for these ions.

## Conclusion

The formation of negative and positive ions upon electron collision with FA clusters embedded in large HNDs was investigated by mass spectrometry. Several homologous cluster ion series could be identified for both charge states. The main features in the cluster anion series resemble those seen upon sputtering of FA cryofilms by fission fragments [16] but several additional homologous series could be identified thanks to the high resolution and dynamic range of the mass spectrometer. The dependence of the anion yield on electron energy confirms that the largest cross section for anion formation is around 22.5 eV, thanks to an indirect process that involves the formation of an electronically excited He^−*^ followed by its migration and electron transfer to the dopant [28]. On the other hand, the stoichiometry of cluster anions other than intact and dehydrogenated FA_n_^−^ differs considerably from anions observed upon electron attachment to bare gas-phase FA clusters at 1 eV [20].

The formation of cations is dominated by the formation of protonated cluster ions [FA_n_H]^+^ but intact [FA_n_]^+^ and a series of [FA_n_-H]^+^ cluster cations are also observed. The abundance of [FA_n_]^+^ decreases with *n* much more rapidly than that of protonated [FA_n_H]^+^ cluster cations.

## Electronic supplementary material

ESM 1 (XYZ 280 bytes)
